# Inequalities between Aboriginal and non-Aboriginal Australians seen through the lens of oral health: time to change focus

**DOI:** 10.1098/rstb.2022.0294

**Published:** 2023-08-14

**Authors:** Angela Durey, Nola Naylor, Linda Slack-Smith

**Affiliations:** ^1^ School of Population and Global Health, University of Western Australia, Perth, Western Australia 6009, Australia; ^2^ Aboriginal Health Strategy, Clinical Service Planning & Population Health, Fiona Stanley Hospital, Murdoch, WA 6150, Australia

**Keywords:** oral health, Indigenous, children, racism, critical reflection, studying up

## Abstract

Inequitable social environments can illustrate changes needed in the social structure to generate more equitable social relations and behaviour. In Australia, British colonization left an intergenerational legacy of racism against Aboriginal people, who are disadvantaged across various social indicators including oral health. Aboriginal Australian children have poorer health outcomes with twice the rate of dental caries as non-Aboriginal children. Our research suggests structural factors outside individual control, including access to and cost of dental services and discrimination from service providers, prevent many Aboriginal families from making optimum oral health decisions, including returning to services. Nader's concept of ‘studying up’ redirects the lens onto powerful institutions and governing bodies to account for their role in undermining good health outcomes, indicating changes needed in the social structure to improve equality. Policymakers and health providers can critically reflect on structural advantages accorded to whiteness in a colonized country, where power and privilege that often go unnoticed and unexamined by those who benefit incur disadvantages to Aboriginal Australians, as reflected in inequitable oral health outcomes. This approach disrupts the discourse placing Aboriginal people at the centre of the problem. Instead, refocusing the lens onto structural factors will show how those factors can compromise rather than improve health outcomes.

This article is part of the theme issue ‘Evolutionary ecology of inequality’.

## 1. Introduction

Aboriginal and Torres Strait Islander (hereafter Aboriginal) Australians are Indigenous peoples who currently make up 3.2% of the overall Australian population [1]. Australia's colonization by the British from 1788 dispossessed Aboriginal peoples of their lands, rights and occupancy as the British assumed authority for policies and practices [2,3]. This led to a legacy of racial segregation that included Aboriginal children, known as the ‘Stolen Generations’ being forcibly removed from their families from 1910–1970 to be assimilated into white Anglo-Australian society [4]. Racism can be defined within the broader concepts of privilege and oppression where different worldviews between social groups manifest through attitudes, beliefs, behaviours, laws, norms and practices [5,6]. More specifically, racism can be a) institutional or systemic (often including structural racism), where ‘the production, control and access to material, information and resources increase the power differentials between racial groups'; b) interpersonal, where a person is treated unfairly in interactions because of race; or c) internalized, where racist attitudes and beliefs are incorporated into one's own worldview [5]. In Australia, racism disproportionately affects Indigenous Australians, who have collective experiences and memory of abuse, discrimination and insensitivity across many sectors including education, health, housing and employment [7,8].

Whiteness is symbolic of privilege and can refer not only to skin colour but also to a racialized social structure reflected in Anglo-Australian cultural dominance, where Indigenous knowledge and culture are subjugated at the level of policy and practice [9,10].

Such policies and practices have led to ongoing intergenerational trauma and grief for Aboriginal peoples, who continue to be disadvantaged across a range of social indicators including health, education, employment and criminal justice [11], and they are reflected in worse health outcomes for Aboriginal compared to non-Aboriginal Australians [12]. In 2008 the Australian government committed to ‘closing the gap’ in Aboriginal disadvantage to improve their health and wellbeing outcomes [13]. While there have been improvements in health including reduced Aboriginal mortality rates [14] and better early childhood education and school retention [15], the ongoing legacy of colonization has led many Aboriginal Australians to mistrust and be reluctant to access mainstream services given their experiences of inequality, injustice and racism [16–18]. Such experiences occur across the range of health conditions commonly impacting Aboriginal people [19].

Mistrust and reluctance are reflected in Aboriginal Australians' experiences in accessing oral health care, the lens through which the evolutionary ecology of inequality will be explored in this paper.

Good oral health is integral to overall health and wellbeing, contributing to quality of life including the ability to eat, speak and socialize without pain, discomfort and embarrassment [20,21]. Good oral health is having no decayed, missing or filled teeth because of dental caries [21]. Although often considered preventable, oral disease is a global public health problem – affecting an estimated 3.5 billion people worldwide [22]. Children living in poverty, socially marginalized and with limited access to dental care are the most affected by oral disease, including in Australia where Aboriginal children have higher rates of dental caries than non-Aboriginal children [23,24].

While often considered preventable, poor oral health (mainly dental caries) and associated preventable hospital admissions (with general anaesthetic) are highly prevalent in Australian children (with higher rates in Aboriginal children) [25,26]. Dental caries are a major contributor to poor oral health, with a causal pathway comprising interaction between fermentable carbohydrate and acid-producing bacteria acting on tooth surface [27]. Broader factors associated with oral disease include high sugar consumption [28], low oral health literacy and limited knowledge around preventing oral disease [29,30]. Oral disease is one of the most prevalent and costly diseases in young children [27,31], where dental services in Australia cost over $9 billion per annum [32]. Current approaches to education, legislation and policy have failed to respond adequately to poor oral health in young Australian children [33].

Oral disease impacts across the life-course and includes dental caries, gum disease and oral cancer, leading to pain, infections and lost productivity. Compromised nutrition resulting from oral disease leads to delayed growth and impaired cognitive and social development, which negatively impact concentration and school participation [27,34]. While the focus of care is mainly on individual treatment and chair-side oral health advice, most oral disease is largely untreated due to limited access to dental care owing to the often prohibitive cost of services and lack of education about preventing oral disease that mainly affects those who have been socially disadvantaged, including Aboriginal children [24,35].

From an evolutionary ecology perspective, the distribution of relational, material and embodied wealth is reflected in group health and wellbeing outcomes. Intergenerational transmission of wealth positively impacts on people's fitness, evolutionary change and capacity to adapt [36]. Those in society who are unable to access such wealth can experience a pervasive sense of risk and uncertainty that is difficult to adapt to and that damages health and wellbeing [37]. Yet, social inequality has been a dominant organizing principle in the hierarchical structure of human society for thousands of years and ‘manifests in unequal access to goods, information, decision making, and power’ ([38]: p. 2). It is ubiquitous in contemporary human society, with damaging social and ecological impacts [39] that are hence reflected in disparities in oral health outcomes between Aboriginal and non-Aboriginal children [23,24].

Wilkinson and Pickett [40] argue that inequality can erode trust and divide society into the 'haves' and 'have-nots' in ways that impact on health and wellbeing, including for Aboriginal Australians. However, inequality can also be mitigated. Wilkinson's [41] analysis of evidence-based research on inequality concludes that the quality of social relations is a central issue facing modern societies that is seen in how conflict over accessing scarce resources is resolved. Societies where inequality between rich and poor is widest, such as the USA, the UK and Australia, reflect dominant hierarches where powerful social groups have access to resources such as health services, education and employment, while groups facing economic and social disadvantage face barriers to accessing the same resources. Societies that have more equality, including the Nordic countries, apply concepts of fairness, reciprocity, trust and mutual aid to practice, where a sense of value is derived from social sources including cooperation rather than power, money and status ([41], p. 286). According to Wilkinson & Pickett ([40]: p. 265) ‘greater equality is the material foundation on which better social relations are built’.

This paper draws on our research into oral health in young Aboriginal children in Western Australia to scrutinize structural and cultural factors that can compromise rather than promote good oral health outcomes. We define structure as social, political and economic factors beyond the control of individuals that can damage their health [42].

## 2. Context

Australia provides a mainly private model of dental care focused on individual treatment rather than prevention at a population level where about 85% dentists are in private practice [43]. The Western Australian government provides public subsidized dental services for eligible adults or children aged 0–4 years whose name appears on their parent's health care or pension concession card. Those needing care are placed on a waiting list. Children aged 5–16 years can access care from the free school dental service provided by the government (see https://www.dental.wa.gov.au/Dental-Services/General-Dental-Service/). Aboriginal Australians can access free dental care through the Aboriginal Community Controlled Health Services established in the 1970s to provide culturally appropriate care [44]. However, many also access private and government clinics for treatment [45].

## 3. Methods

This paper revisits our published qualitative research to inductively synthesize key findings focusing on structural issues impacting oral health in young Aboriginal children. Three discrete qualitative research projects on oral health in Aboriginal children were published in 2016, 2017 and 2021 and specifically sought the perceptions and experiences of Aboriginal Health Workers, Aboriginal parents and dental practitioners on factors that contributed to or impeded good oral health in this cohort [46–48]. These are summarized in table 1. Findings from these projects identified the role that structural issues play in Aboriginal children's oral health, including the prohibitive cost of services, a model of care focusing on individual treatment without addressing the social context of families' lived experience and racism from service providers where Aboriginal participants often felt judged about the state of their children's poor oral health, which led to their reluctance to attend dental services for follow-up care [46–48].

**Table 1.** Summary of projects related to oral health in Aboriginal children in Perth, Western Australia.

published paper	data collection	research questions	analysis—key themes	role of structural issues
Aboriginal Health Worker perceptions of oral health: a qualitative study in Perth, Western Australia [46]	11 interviews, 4 focus groups with Aboriginal Health Workers—36 Participants	What helped or prevented good oral health in participants' communities?	**Barriers**: current models not meeting demand; limited opportunity to access services; cost; emergency dental care; racism from some service providers. **Enablers**: schools’ dental service.	Mostly private model of care at prohibitive cost; focus on treatment rather than prevention; racism from some service providers
Oral health in young Australian Aboriginal children: qualitative research on parents' perspectives [47]	9 group discussions with 52 parents/carers conducted across 10 sites	Participants' perspectives on dental visits. Role of diet in oral health? Improvements to oral health in Aboriginal families?	**Barriers**: cost of dental services and providing healthy diet for families (processed foods cheaper); competing demands on limited budgets; negative judgements from service providers; reluctance to attend services; emergency dental care. **Enablers**: oral health is important; schools' dental service.	Current models of oral health not meeting families' needs; high cost of dental services; prohibitive cost of providing healthy diets for families; racism
Dental professionals' perspectives working with Aboriginal children in Western Australia: a qualitative study [48]	Interviews with 12 dental professionals experienced in working with Aboriginal children	What helped or hindered providing dental care to Aboriginal children?	Social determinants of oral health beyond remit of clinical practice to address; heavy marketing of sugar products; frustration with focus on individual treatment rather than prevention; lack of education and professional development in caring for Aboriginal children	Ignoring social determinants of oral health in individual model of dental care; dental system's focus on individual treatment rather than prevention at population level

We were guided in our interpretation by the concept of ‘studying up’ to reflect more closely on structural issues to better understand their role in the evolutionary ecology of inequality. ‘Studying up’ was a concept used by anthropologist Laura Nader [49] in the 1970s, who argued for bringing attitudes and practices of the powerful under closer scrutiny around, for example, issues of accountability in relation to social inequality. 'Studying up’ addresses inequality by redirecting questions to ‘study the culture of power rather than the culture of powerlessness' ([49], p. 289) and by calling to account powerful institutions and governing bodies for their role in undermining rather than promoting good health outcomes. While Nader [49] highlighted the importance of studying the powerful, Gusterson [50] questioned the ongoing tendency in anthropology to study the disadvantaged. He noted that ‘the cultural invisibility of the rich and powerful is as much a part of their privilege as their wealth and power, and a democratic anthropology should be working to reverse this invisibility’ ([50] p. 115). While Nader ([49] p. 302) acknowledged that powerful elites are often difficult to engage as they are ‘out of reach on a number of different planes: they don't want to be studied; it is dangerous to study the powerful; they are busy people’, some more recent research has been successful in this area. Relations of power have been examined in various contexts including a nuclear weapons laboratory in California [51]; Wall Street financial institutions [52]; the fairness of an algorithmic system in pretrial risk assessment in American criminal law [53] and feminist reflections on interviewing elite men [54]. Studying the powerful can help identify how hegemonic relations are constructed and reproduced [55]. *La Cascada Declaration* [35] and other international work [56] acknowledge the need to shift focus by identifying a crisis in dentistry, centred on treating disease at an individual, biomedical level rather than on prevention.

Nader appealed for a critical repatriated anthropology that would shed light on processes of exploitation and domination by refocusing the anthropological lens on the cultures of the powerful. As Nader argued, ‘If one's pivot point is around those who have responsibility by virtue of being delegated power, then the questions change’ ([49], p. 290). We use this concept to interrogate findings from the three qualitative research projects mentioned above to examine the nature of structural problems and just where they lie.

## 4. Findings

Findings from the three papers (Durey *et al*. 2016; 2017; 2021) [46–48] have been summarized in table 1.

Analysis focused on ‘studying up’ and identified structural factors underpinning poor oral health in Aboriginal families. Our findings supported other research evidence indicating how such factors impeded progress in improving oral health outcomes in Aboriginal children [23]. Structural factors included the corporate determinants of health promoting the marketing of sugar products to influence consumption despite their damaging effects on oral health outcomes [57,58] and the current model of dental care. Dental services focus mainly on individual treatment and have done little to prevent disease at a population level. Individual treatment fails to take into account the social context in which Aboriginal children's lives are embedded that impact on decisions related to oral health [35,59]. Instead, Aboriginal participants often felt blamed for the state of their or their children's oral health [46,47].

Focusing on individual responsibility is underpinned by a neoliberal political ideology that ‘mandates shifts from governmental responsibility to individual responsibility’, including for health ([60], p. 17). There is a tendency to penalize individuals for ‘non-compliance’ with health advice and to blame them for ‘personal failure’ related to their poor heath [61]. This approach removes the focus from examining the role of structural elements in constraining individuals to make optimum oral health choices [62–64]. For example, our findings supported other evidence that racism against Aboriginal Australians, including in health services, is harmful to their health and wellbeing [16,65] and leads to Aboriginal people's reluctance to attend health services [46,47]. If discriminatory policies and practices towards Aboriginal people are normalized, go unnoticed and unexamined by policymakers and service providers, ignoring the harm they can cause, then health inequalities between Aboriginal and non-Aboriginal Australians are likely to persist. In the words of a previous Australian Prime Minister, Paul Keating, in his 1992 Redfern Speech: ‘*The starting point might be to recognize that the problem starts with us non-Aboriginal Australians. It begins, I think, with the act of recognition’* [66].

If we take racism as an example of a structural issue impacting on choice, our evidence suggests that more is needed in health care than just delivering evidence based interventions [67]; instead, structural issues underpinning intergenerational social and historical contexts of Aboriginal people's lives need to be understood for their role in informing health choices. Critical self-reflection by non-Aboriginal policymakers and health providers on whether their beliefs and assumptions about Aboriginal people are discriminatory or demeaning is imperative. The objective of such reflection is to avoid projecting any unconscious bias onto Aboriginal people that could negatively impact on their health and wellbeing [62,68].

In Australia, whiteness—as in white Anglo-Australian racial and cultural dominance—is the norm, which is often assumed and rarely interrogated by those privileged in this system. Whiteness is the benchmark against which differences from that norm are measured valued and judged [2]. Being white in colonized countries such as Australia confers structural advantage that is usually invisible to those who are white (but not to Aboriginal Australians) and this advantage operates through a set of cultural practices, such as education, health, employment and the criminal justice system [69]. These cultural practices shape both the lives of those who are privileged as well as those who are marginalized [70].

While whiteness can refer to skin colour, it also reflects a racialized social structure where, in a dominant western biomedical health context, Aboriginal knowledge, beliefs and values are ignored at the level of policy and practice [71]. Researchers and health providers from different ethnic backgrounds who are educated or trained in the predominantly western biomedical care may, according to Kowal, ‘willingly or unwillingly, knowingly or unknowingly, participate in a racialised social structure’ that privileges knowledge and practices associated with the dominant Australian culture ([71]: p. 341). A critical analysis of whiteness, and the power and privilege it provides, is an important step in interrogating the discourse that historically has positioned white colonizers at the top and Aboriginal Australians at the bottom of the hierarchical racial structure [72]. Thus, race can be seen as a structuring or organizing principle in social relations that is underpinned by relations of power [2,9]. Currently, Aboriginal Australians are often still positioned at the centre of the problem, shifting the gaze away from interrogating structural factors. While there have been improvements in Aboriginal health, it is important to recognize that privilege often remains ‘invisible, natural, normal and unmarked’ ([2]: p. 183) despite it being, as Pease [68] suggests, the flip side of discrimination that needs to be considered.

The trope of ‘I treat everyone the same’ is often heard by health professionals and worn as a badge of honour. The concept of cultural safety is an effort to contravene such tropes [73,74]. McGibbon *et al.* [75] argue that nursing knowledge is steeped in an ethos of treating everyone equally, one-size-fits-all, with the assumption that, in a colonized country, the dominant culture, knowledge, experience and values are true and applicable across all cultures. However, evidence suggests that health providers do not treat everyone the same, based on their beliefs about the socioeconomic and cultural circumstances of certain social groups. For example, in addition to our own research into oral health in Aboriginal children, Boffa's [76] research into medical practitioners suggests that any negative assumptions that doctors held about Aboriginal Australians increased the likelihood of offering fewer options for treatment and appropriate care compared to that offered to non-Aboriginal Australians.

According to our findings, good oral health mattered to Aboriginal participants, challenging the assumption that if oral health is poor, then Aboriginal people are somehow to blame for not complying with evidence-based messages around eating a healthy diet low in sugar, maintaining their oral hygiene and going for regular dental check-ups [46,47]. While all these activities are important, our findings reflect the context of Aboriginal participants' lived experience and indicate that structural factors such as racism and the cost of services constrain Aboriginal participants in making optimum choices about their oral health and potentially compromising good oral health outcomes [46,47]. The WHO Commission on Social Determinants of Health ([77], p. 1) argues that structural conditions inform lived experience and are responsible for significant health inequities where:

…unequal distribution of health-damaging experiences is not in any sense a ‘natural’ phenomenon but is the result of a toxic combination of poor social policies and programmes, unfair economic arrangements, and bad politics.

Yet the system itself—including policymakers, health providers and the sugar industry—overlooks and seems reluctant either to acknowledge that it may be part of the problem or to examine its own role in potentially compromising rather than improving oral health for Aboriginal people, illustrating Wisniewski's ([78]: p. 5) concept of ‘the averted gaze’ and thus maintaining the status quo [9].

Studying up and refocusing the lens to include structural factors when studying oral health in Aboriginal children could provide a deeper understanding of the evolutionary ecology of inequality and how structural factors produce undesirable social and health outcomes [49,79]. This could lay the foundation for a committed focus on health equality, with more robust forms of accountability, if policymakers, researchers and health providers adopt a critically reflective approach to practice and examine their own assumptions about certain social groups and how those assumptions can impact on their practice and their patients' health outcomes [9]. This leads us to consider the consequences of not studying powerful groups if we are serious about reducing health inequalities. To contribute to creating a more equitable society, different questions need to be asked that focus on, for example, structural accountability around delivering high-quality, equitable oral care to Aboriginal Australians. For example, if measures of good oral health are the absence of tooth decay, the belief that dental caries are preventable (at least in theory), and that evidence-based health messages to maintain oral health include eating a healthy diet with a low sugar intake, tooth-brushing and regular dental visits, then questions are raised about how structures such as models of health service delivery are supporting the context of Aboriginal people's lives and their capacity to implement these messages [35,80].

According to Wilkinson ([41], p. 235), responses to inequitable social environments are not predetermined from an evolutionary perspective as they can also guide us in what needs to change in the social structure to generate a better quality of social relations and social behaviour. In colonized countries such as Australia there is greater inequality between those who are privileged based on race and those who are disadvantaged, with damaging psychological, physiological and pathological effects of social prejudice [16,17,41]. According to Wilkinson [41], a key issue affecting the health and wellbeing of hierarchical societies like the USA, the UK and Australia is the quality of social relations within and between social groups. In colonized countries greater inequality in society is associated with poor-quality social relations and concomitant damaging health effects of increased chronic stress for those who are socially disadvantaged [17,18,65]. Knowing this offers an opportunity to learn from countries with greater equality and better health and wellbeing outcomes and to consider the kind of society we want to develop where oral health inequalities between Aboriginal and non-Aboriginal Australians are reduced and ultimately eliminated altogether [40]. Shifting the focus away from blaming Aboriginal people for poor oral health to refocusing the lens on the role of the broader structural context in informing choices can help inform how the problem of poor oral health in Aboriginal families can be more effectively addressed. While changes are occurring in Australia, including the Close the Gap initiative [15], ensuring that they are embedded and sustained in policies and practice requires bi-partisan political will and a concerted commitment to creating a more equitable and just society.

## 5. Conclusion

‘Studying up’ is a concept we have used to highlight the role that structural factors play in the evolutionary ecology of inequality in Aboriginal children's oral health and it offers an opportunity to reconsider the focus of oral health care in this context. A 'one-size-fits-all' approach focused on individual treatment has done little to improve oral health outcomes at a population level. Instead, a more equitable approach is called for, where policies and practices are decolonized and centre on Aboriginal families' voices and lived experiences. This requires diverting conventional discourse on oral health care for Aboriginal children away from a focus on marginalized social groups to critically reflecting instead on the role and power of dominant white structures that discriminate against Aboriginal Australians. Undertaking such critical analysis offers an opportunity for white institutions to reposition institutional policies and practices to ensure they improve rather than compromise oral health outcomes for Aboriginal children at a population level.

## Data Availability

This article has no additional data.
